# Long-term outcomes in nephropathic cystinosis: a review

**DOI:** 10.1007/s00467-025-06790-6

**Published:** 2025-05-14

**Authors:** Hayley E. Chang, Mahin S. Hossain, Chris Song, Narayana Surampudi, Galina Nesterova, William A. Gahl

**Affiliations:** 1 Cognitive Neurophysiology Lab, Del Monte Institute for Neuroscience, University of Rochester School of Medicine and Dentistry, Rochester, NY USA; 2 Kuan Hong Wang Lab, Del Monte Institute for Neuroscience, University of Rochester School of Medicine and Dentistry, Rochester, NY USA; 3 Section on Human Biochemical Genetics, Medical Genetics Branch, National Human Genome Research Institute, National Institutes of Health, Bethesda, MD 20892 USA; 4 Rush University Medical College, Rush University, Chicago, IL USA; 5 NIH Biomedical Translational Research Information System (BTRIS), Clinical Center, National Institutes of Health, Bethesda, MD USA; 6 PPD, Thermo Fisher Scientific, Waltham, MA USA

**Keywords:** Cystinosis, Fanconi syndrome, Cysteamine, Nephropathic, Complications, Cystine, Multisystemic disorder

## Abstract

Nephropathic cystinosis is a lysosomal storage disease due to biallelic pathogenic variants in the *CTNS* gene encoding the cystine transport protein cystinosin. Dysfunction of cystinosin results in the intralysosomal accumulation of the disulfide cystine, which crystallizes in some tissues and damages many parenchymal organs. Fanconi syndrome is the first presenting sign with all the features of generalized proximal tubular dysfunction. The natural history of cystinosis includes multisystem complications, the most prominent being glomerular failure at 9–10 years. If a kidney transplant prolongs life, other complications occur, with variable frequencies. Some of the most common are hypothyroidism, a distal vacuolar myopathy, pancreatic exocrine and endocrine insufficiency, male hypogonadism, and idiopathic intracranial hypertension. Cystinosis is diagnosed biochemically by measuring the cystine content of leucocyte and molecularly by identifying pathogenic variants in *CTNS*. Prenatal diagnosis is available. Treatment consists of replacement of kidney tubular losses, symptomatic management of systemic complications, and specific therapy directed at the basic defect, i.e., lysosomal cystine accumulation. This involves the free thiol cysteamine, which can deplete approximately 95% of the lysosomal cystine content. Oral cysteamine therapy has extended the time to kidney failure by approximately 7 years (to a mean of 16 years) and mitigates or prevents late complications of the disease. In addition, cysteamine eyedrops can dissolve corneal cystine crystals within months. Nevertheless, the mean age at death for individuals born between 1985 and 1999 has been 29 years, and earlier diagnosis by newborn screening, treatment with more palatable cystine-depleting agents, and trials with gene therapy are critical current pursuits.

**Graphical abstract:**

**Figure:**
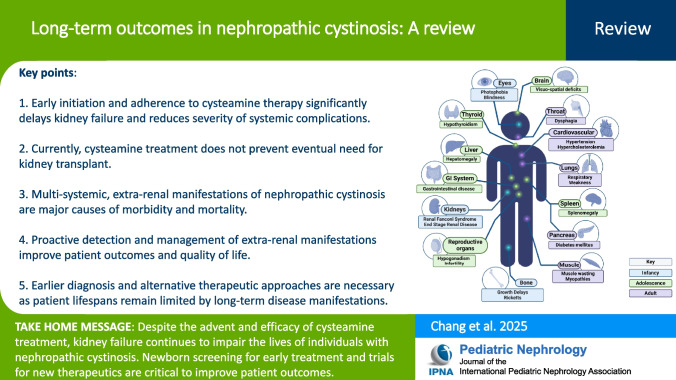
A higher resolution version of the Graphical abstract is available as  Supplementary information

**Supplementary Information:**

The online version contains supplementary material available at 10.1007/s00467-025-06790-6.

## Background

Nephropathic cystinosis is a rare, autosomal recessive lysosomal storage disease with an incidence of 1:100,000 to 1:200,000 live births [1]. Crystal accumulation in cystinosis was first described by Abderhalden in 1903 and the glucose wasting of cystinosis was recognized in 1931 by Dr. Guido Fanconi [2], after whom the Fanconi syndrome was named. The intracellular storage of cystine in fibroblasts was demonstrated by Schneider et al. in 1976 [3]. In 1982, the basic defect in cystinosis was elucidated as deficient or absent transport of cystine out of lysosomes [4]; heterozygotes for cystinosis have half the cystine-transporting capacity [5] but never have any clinical manifestations of the disease [1, 2]. Cystinosis stands as a prototypic disorder for a subgroup of lysosomal storage diseases caused by defective lysosomal transporters [2].

While three forms of cystinosis have been described, the disorder displays a continuum of clinical severity inversely related to the amount of residual cystine-transporting activity. The most common and severe form, infantile or nephropathic cystinosis, constitutes roughly 95% of all cases and is associated with kidney failure at 9–10 years of age [2]. The mildest form of cystinosis is termed “adult” or “ocular,” since the clinical manifestations are limited to the occurrence of corneal cystine crystals [2]. A juvenile, adolescent, or intermediate form manifests kidney dysfunction in the second or third decade of life and is likely underdiagnosed. These patients may first present with ocular discomfort and photophobia and be found to have mild glomerular and/or tubular impairment. They can progress to kidney failure, some requiring kidney transplants [6]. More research is needed to understand the nuanced differences between the early and late forms of the disease.

### Genetics

All forms of cystinosis are caused by biallelic, pathogenic variants in the *CTNS* gene located on chromosome 17p21 and identified in 1998 [7]. *CTNS* is 26 kb in length, with 12 exons that produce the 367-amino acid lysosomal transport protein, cystinosin. This cystine transporter has seven transmembrane domains and two lysosomal targeting motifs; it is ubiquitously expressed. Well over 100 different mutations have been documented in cystinosis [8, 9]. The most common mutation is a deletion that encompasses the first 10 exons of the gene along with portions of the *CARKL* gene upstream of *CTNS*; this founder deletion occurs primarily in individuals of European descent and likely arose in Germany in approximately 700 AD [10]. Specific mutations have been reported in intermediate cystinosis patients [11] and the 928 G>A (G197R) mutation in *CTNS* is associated with ocular cystinosis [12].

### Diagnosis

The mean age of diagnosis is approximately 14 months [1, 2]; it often depends upon a high index of suspicion engendered by the finding of failure to thrive and Fanconi syndrome. A definitive diagnosis is made by finding increased levels of cystine in polymorphonuclear leucocytes. Normal levels are < 0.2 nmol of half-cystine/mg of protein; affected individuals have values of 3–25 nmol half-cystine/mg of protein and heterozygotes have values up to 1.0 [2]. Corneal crystals are also pathognomonic of the disease. The diagnosis can be confirmed by *CTNS* molecular analysis. Prenatal diagnosis can be achieved by assaying cystine in amniotic fluid cells or chorionic villus samples [2] or by performing *CTNS* sequencing on these tissues.

### Initial presentation: Fanconi syndrome

Nephropathic cystinosis generally presents within the first 6 to 12 months of life with signs of proximal tubular dysfunction, i.e., Fanconi syndrome. This involves failure to reabsorb water, minerals, electrolytes, amino acids, carnitine, and other small molecules including low molecular weight proteins. Young children can have daily urine volumes of 2–6 L; excessive thirst and dehydration, failure to thrive, and poor growth comprise the first signs of the disease. Due to tubular wasting of phosphate, hypophosphatemic rickets develops, with skeletal deformities and delayed ambulation. Cystinosis remains the most common cause of Fanconi syndrome in children.

Treatment of Fanconi syndrome requires fluid, electrolyte, and mineral replacement [13]. Access to liquids, preferably containing calories, is essential. With viral infections involving vomiting and diarrhea, early parenteral fluid replacement is critical. Potassium wasting is universal in cystinosis prior to transplantation and requires supplementation; acidosis due to bicarbonate losses is treated with citrate or bicarbonate. Hypocalcemia can cause tetany and require calcium supplementation. Children with cystinosis often crave salt and sodium should not be restricted. Interestingly, they also crave ketchup and spicy foods like jalapeno peppers. Hypophosphatemic rickets is treated with sodium or potassium phosphate along with calcitriol to enhance gastrointestinal absorption of phosphate.

### Glomerular involvement

In cystinosis, glomerular damage likely begins early in life. However, the functional reserve of the kidney compensates, and serum creatinine does not increase noticeably until approximately 5 years of age, even if untreated. In the absence of cystine-depleting therapy, progressive glomerular damage results in kidney failure at 9–10 years of age [14]. Prior to the availability of dialysis and kidney transplantation in the late 1960s, this was associated with death. Kidney allografts do very well in cystinosis patients, and cystine storage does not recur in the transplanted kidney [2]. Nevertheless, many patients eventually require additional allografts. Biopsy of a donor kidney may contain crystals, but these are within invading host cells. The normal transplanted kidney does not impede the accumulation of cystine in other tissues of the body. In addition, kidney transplantation requires standard immune suppression, which has been shown to increase the risk for hematological malignancies in large observational studies of patients with kidney failure (KF) [15]. One consideration surrounding a kidney allograft is whether to remove the native kidneys; if some function remains, there can be a loss of potassium, water, and other small molecules, endangering the health of the donor kidney.

### Treatment with cysteamine

**Fig. 1:**
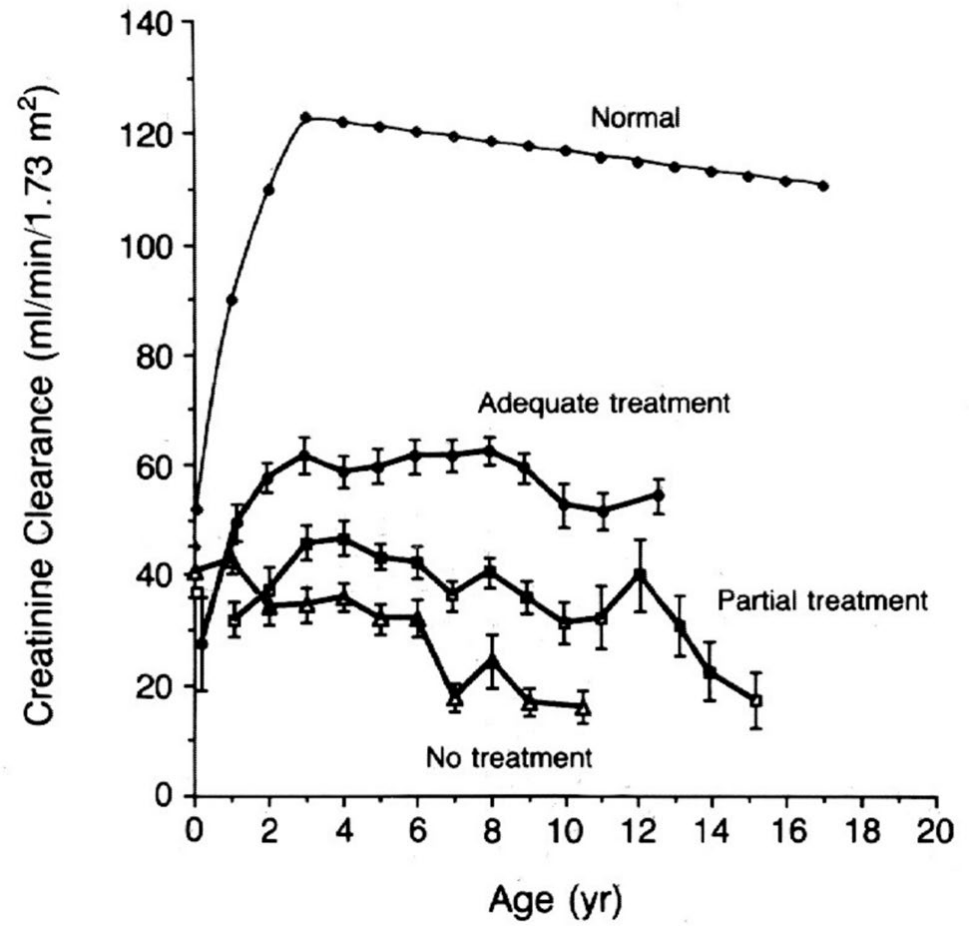
Mean creatinine clearance as a function of age in normal subjects, 17 patients with cystinosis who had received adequate treatment with cysteamine, 32 who had received partial treatment, and 67 who had not received treatment standard errors are shown for the three groups of patients with cystinosis [20]

Oral cysteamine therapy targeting the basic defect is a life-saving treatment. This free thiol passes through the plasma and lysosomal membranes by virtue of its uncharged amine group and participates in a disulfide interchange reaction with cystine to produce cysteine and cysteine-cysteamine mixed disulfide, both of which, unlike cystine, can exit the cystinotic lysosome [16]. In 1976, Thoene and Schneider showed that cysteamine reduced the cystine content of cystinosis fibroblasts in culture [17]. There followed an international clinical trial of oral cysteamine, using a historical control cohort of patients treated with a placebo in a previous, unsuccessful trial of ascorbic acid. The cysteamine clinical trial and subsequent reports demonstrated the safety and efficacy of oral cysteamine with respect to the maintenance of glomerular function and growth [18, 19]. In 1993, Markello et al. described an intent-to-treat analysis of all cystinosis patients evaluated at the NIH Clinical Center between 1960 and 1992 [20]. They documented increasing creatinine clearance in the first 3 years of life in patients well-treated with cysteamine, modest maintenance of glomerular function in moderately well-treated individuals, and a monotonic loss of kidney function in patients who never received oral cysteamine (Fig. 1). In addition, a nearly normal growth rate was achieved with excellent compliance to cysteamine therapy. Oral cysteamine therapy was approved by the FDA as Cystagon^®^ in 1994. The recommended dose is 60–90 mg/kg/day or 1.3–1.95 g/m^2^/day, divided Q6H. A delayed release form of cysteamine (Procysbi®) is available as Q12H therapy.

The cornea is avascular, so oral cysteamine does not reach the corneal cells that store cystine and form corneal crystals. Consequently, cysteamine needs to be delivered topically. A solution of 0.1–0.5% cysteamine hydrochloride can dissolve the corneal crystals of cystinosis in months to years if administered several times per day [21, 22].

## Cystinosis as a multisystemic disease

Although the kidney is affected first and foremost in cystinosis, many other organ systems are involved. Late complications include a distal vacuolar myopathy [23, 24] and its concomitant swallowing impairment [25, 26], pancreatic endocrine and exocrine insufficiency [2, 27], cerebral atrophy and basal ganglia and periventricular calcifications [28], deficient sweat production [29], ocular involvement such as band keratopathy and retinal blindness [30], idiopathic intracranial hypertension [31], and coronary artery calcification [32]. Males have low testosterone and are infertile due to testicular fibrosis and atrophy [33], but females retain ovarian function and have successfully delivered normal babies [34]. Without early and consistent treatment, cystinosis patients are likely to experience at least one significant complication of the disease by the age of 30 [35].

### Bone

**Fig. 2:**
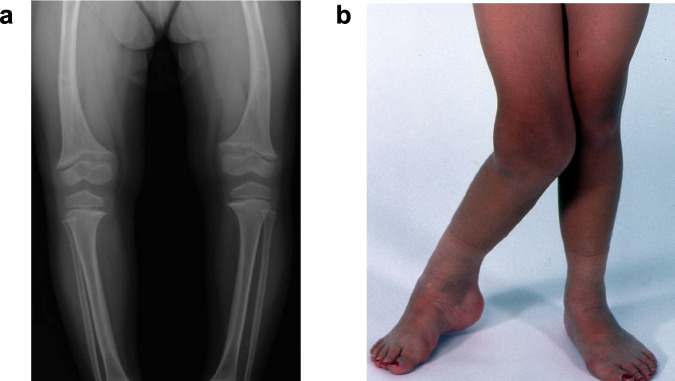
Bone abnormalities in cystinosis. **a** Hypophosphatemic rickets with characteristic bowing, metaphyseal widening, epiphysial fraying, and osteoporosis. **b** Deformity due to kidney osteodystrophy

Cystinosis-associated metabolic bone disease (CMBD) results from a combination of Fanconi syndrome leading to hypophosphatemic rickets (Fig. 2a) and nutritional deficiency, hormonal disturbances, myopathy, and chronic kidney disease.

Fanconi syndrome causes phosphate, bicarbonate, and calcium wasting, metabolic acidosis, hypophosphatemic rickets, growth retardation, osteomalacia, bone pain, skeletal deformities (genu valgum and scoliosis), fractures, and reduced bone mineral density [9]. Fractures are frequent, particularly in the long bones and vertebrae; radiographs demonstrate abnormal skeletal microarchitecture and signs of metabolic bone disease [36, 37]. In a French study of 10 patients aged 10–35 years, 70% had a bone complication (fracture, deformity, or pain) during adolescence or young adulthood [38]. Furthermore, patients had osteomalacia and osteopenia with significant alterations in cortical thickness and total volumetric bone mineral density at the tibia and radius when compared to controls [39]; cortical bone deficits are more pronounced than those in trabecular bone. In another study of 30 NIH patients (mean age of 20 years), Florenzano et al. found bone mineral density was reduced at all assessed sites [37]. Large proportions of patients displayed bone symptoms, i.e., one or more fractures of the long bones (27%), incidental vertebral fractures unrelated to transplant status (32%), long bone deformities or bowing (64%), and scoliosis (50%). Some patients with kidney failure develop kidney osteodystrophy (Fig. 2b).

Glucocorticoid use, which was common in the past as part of an anti-rejection regimen for cystinosis patients with a kidney allograft, is used much less since steroid-free regimens are available. Glucocorticoids worsen kidney osteodystrophy and skeletal abnormalities [40]. Other systemic manifestations of cystinosis such as muscle wasting/myopathy, male hypogonadism, and hyperleptinemia [41] also worsen bone health by decreasing bone mineral density and strength.

Recent studies have highlighted several key differences in the pathophysiology of bone disease in cystinosis compared to other CKDs. For example, in CKD, levels of FGF23, a key regulator of phosphate metabolism, increase as kidney function declines, contributing to phosphate excretion and secondary hyperparathyroidism. However, in cystinosis, FGF23 levels remain paradoxically low despite persistent hypophosphatemia from renal Fanconi syndrome [42, 43]. Corroborating these reports, Ewert et al. demonstrated patients with cystinosis exhibit significantly lower FGF23 levels than CKD controls across all CKD stages, suggesting phosphate-driven regulation rather than kidney dysfunction [42]. Florenzano et al. confirmed that lack of hyperphosphatemia in cystinosis prevents FGF23 elevation, delaying the onset of secondary hyperparathyroidism [43]. In another study, cystinosis patients with advanced CKD exhibited suppressed FGF23 levels, alongside high levels of bone turnover markers, highlighting a distinct mineral imbalance different from other forms of CKD and underscoring the need for cystinosis-specific management strategies [36].

In addition, unlike CKD patients who typically exhibit elevated sclerostin levels due to declining renal clearance, patients with cystinosis demonstrate lower sclerostin, particularly in early CKD stages. This may be due to persistent phosphate wasting and altered bone remodeling, which influences osteocyte signaling [36, 42]. After kidney transplantation, cystinosis patients demonstrate gradual increases in sclerostin, reflecting improvements in kidney function and bone metabolism. However, despite normalization of renal clearance, persistent skeletal abnormalities suggest that intrinsic osteoblast/osteoclast dysfunction, rather than sclerostin regulation alone, contributes to bone disease in nephropathic cystinosis [36].

Although DEXA scans are widely used in CKD-MBD, their role in cystinosis is significantly limited due to growth delays and DEXA scans’ limitations in assessing bone disease or predicting fracture risk in cystinosis [44]. DEXA scans measure areal bone mineral density (BMD) which is unable to distinguish between osteomalacia (mineralization defect) and osteoporosis (bone mass loss). Given the complex nature of CMBD, which involves both impaired mineralization and cortical bone thinning, DEXA results may not fully capture the underlying pathology. Because of growth delays in cystinosis, especially in pediatric patients, DEXA results, when not adjusted for body size, may underestimate BMD in cystinosis patients. Finally, in contrast to other CKDs, fractures in cystinosis patients are not correlated with low DEXA scores, suggesting that factors such as bone geometry, cortical bone thinning, and intrinsic bone metabolic defects play a greater role in fracture risk than BMD alone. Regular X-rays, especially of the knees and wrists, are preferred for assessing bone deformities. Furthermore, high-resolution peripheral quantitative computer tomography (pQCT) is able to differentiate between cortical and trabecular bone and may be more informative in cystinosis [44].

Early and consistent monitoring of serum levels of phosphate, calcium, alkaline phosphatase, and parathyroid hormone are necessary [9]. Serum phosphate levels may appear normal due to bone resorption even in the setting of kidney phosphate wasting, so serum alkaline phosphate levels are a better indicator of rickets status. Vitamin D and phosphate supplementation remain cornerstones of management [40]. Physical therapy and diligent cysteamine therapy can help slow the progression of bone disease, but neither can completely prevent bone abnormalities [45].

### Growth

At birth, neonates are typically of normal size, but infants usually fall off their height curves at 6–9 months of age, reaching the third percentile for height by 1 year of age. Without treatment, children with cystinosis grow at 50–60% of the normal rate [46]; bone age is generally delayed by 1–2 years and this delay is more pronounced than what is observed in other children with KF alone. Early adherence to oral cysteamine and phosphate replacement therapy allows children with cystinosis to achieve a nearly normal growth rate, and many have height profiles following the 10th–25th percentiles for age [47]. These treatments, however, do not provide catch-up growth. Supplementation with human growth hormone (GH) in children can improve final height, but normalization of stature remains uncommon, and treatment should be started early in the course of KF, before puberty, and before kidney transplant [45, 48]. Interestingly, individuals with cystinosis are not typically GH deficient, although some data have shown altered timing of a GH peak after glucagon stimulation, possibly indicating subclinical alterations in GH secretion [49, 50]. Nevertheless, GH therapy should be initiated only after consultation with subspecialists with direct clinical experience in treating cystinosis or growth delay [51].

### Dental issues

A significant delay in dental development, apparent at about 9 months, is part of the spectrum of generalized growth delay seen in cystinosis [52]. Other dental issues include taurodontism prevalence, caries, enamel defects, intraoral soft tissue findings, hypoplastic and pitted enamel, dentin weakness, poorly defined lamina dura, enlarged pulp chambers, and shortened dental roots. As a result, regular dental exams are recommended for those with cystinosis.

### Endocrine and glandular complications

Nephropathic cystinosis impairs both endocrine and exocrine gland functions. Endocrine organs affected include the thyroid, pancreas, gonads, adrenal glands, and parathyroid glands.

#### Hypothyroidism

In cystinosis, the thyroid gland’s parenchymal accumulation of cystine disrupts follicular cell function [9]. Primary hypothyroidism occurs in up to 75% of patients by the second decade of life [45, 53]; TSH levels can be 100 times normal. A large retrospective study demonstrated that untreated individuals experience hypothyroidism at a median age of 10 years, whereas those receiving long-term cysteamine therapy often retained thyroid function into adulthood [54]. In fact, initiation of cysteamine therapy has shown significant benefits by delaying the onset of hypothyroidism and, if begun early enough, avoiding it altogether [9, 53]. Levothyroxine treatment effectively restores the euthyroid state.

#### Diabetes mellitus

Diabetes mellitus (DM) is a prominent complication of cystinosis, particularly in post-transplant patients, with an incidence as high as 50% in some cohorts [55, 56]. This results from progressive cystine accumulation in beta cells of the pancreas, impairing insulin secretion. A longitudinal study by Robert et al. demonstrated a steady decline in first-phase insulin release and increasing rates of impaired glucose tolerance, even in young adults [56]. Notably, corticosteroid use during transplantation accelerates hyperglycemia, but long-term insulin dependence correlates more closely with the underlying cystinosis pathology [54, 56]. The combined effects of cystine storage in pancreatic beta cells and immunosuppressive medications, such as tacrolimus, exacerbate DM risk, with diminished insulin and C-peptide secretion observed in both pre- and post-transplant patients [55].

#### Pubertal and gonadal function

Delayed puberty and gonadal dysfunction are common in cystinosis patients, especially in those not treated with cysteamine. Male patients display primary hypogonadism, characterized by elevated gonadotropin levels and subnormal testosterone, as well as azoospermia that causes infertility later in life [33, 57, 58]. In 18 males with cystinosis evaluated for serum hormone levels, both LH and FSH were elevated in most cases, consistent with hypergonadotropic hypogonadism [57]. Male patients often have sparse body hair and smaller testes than age-matched peers; some never reach full Tanner staging. Most male patients can achieve normal secondary sex characteristics, although in their early twentiess [33, 57].

Female patients generally have normal ovulatory cycles and gonadal development. There can be delays in puberty, with menarche occurring later than average [59]. Hormone levels like FSH, LH, and estradiol may be low in adolescence but usually normalize in adulthood. Menstrual irregularities have been documented in later stages of the disease.

#### Adrenal and parathyroid gland involvement

Secondary hyperparathyroidism, related to chronic kidney disease and post-transplantation states, can compound skeletal issues such as rickets and osteomalacia [53]. Adrenal insufficiency has been noted, particularly in advanced cases [45]. These conditions often require vigilant monitoring and supplementation with hormones and electrolytes [40].

#### Other glandular defects

Pancreatic exocrine insufficiency, presenting with steatorrhea, occurs in a fraction of post-transplant patients [34, 35] and is treated with pancreatic enzyme supplements. Sweat and tear glands are damaged as well, creating poor heat tolerance and dry eye, respectively [13, 29].

### Reproductive health—fertility and pregnancy

Male fertility is a major problem in cystinosis. Testicular ultrasound revealed signs of obstruction in 67% of patients [57]. Histologic examination showed atrophy and lysosomal cystine overload in peripheral Leydig and Sertoli cells of the testes, suggesting that while spermatogenesis is impaired peripherally, it may remain intact in portions of the central testis [33, 57, 59–61]. Most testes examined also had tubular atrophy, with fibrosis in seminiferous tubules likely contributing to smaller testicular size [33, 57, 59]. Early initiation of cysteamine therapy can delay infertility onset but does not prevent long-term testicular degeneration [57]. Patients should routinely be assessed for gonadal hormones and testicular volume in adolescence [33, 57, 62]. Early discussions of pubertal delays and fertility preservation measures like cryogenic sperm banking before puberty are recommended [63]. Some patients with cystinosis have conceived children using assistive reproductive technologies, but proper genetic counseling and planning remain important.

Despite having delayed puberty, females are generally fertile. Women with cystinosis who plan on becoming pregnant should have comprehensive prenatal counseling, which may include genetic counseling prior to conception [59, 62]. Kidney function should be carefully monitored throughout pregnancy. Cysteamine has the potential to be teratogenic, though there are no definitive guidelines for cessation of treatment. Cesarean delivery is more common in women with cystinosis. In a European case study, the pregnancy success rate was 68.4%, with 5 unviable pregnancies and one neonatal death at 24 weeks [64]. The live births were delivered by C-section [59]. Preeclampsia complicated 46.7% of these births [59]. Although there is concern regarding breastfeeding while taking cysteamine, the concentrations of cysteamine in breast milk are calculated to be very small.

### Ocular involvement

**Fig. 3:**
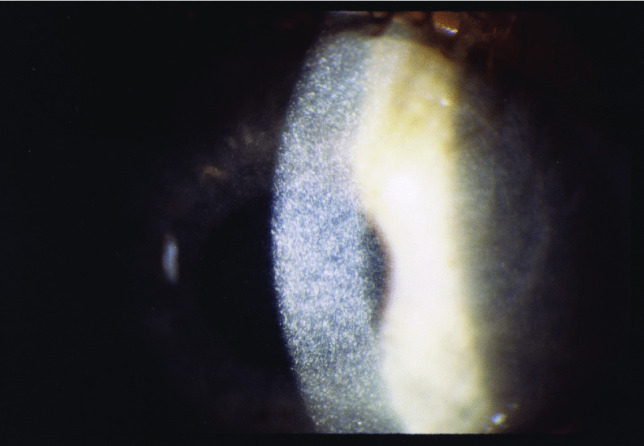
Slit lamp photograph of the cornea of a 3-year old with cystinosis showing myriad refractile cystine crystals

A hallmark of cystinosis is the occurrence of cystine crystals in the cornea, visible on slit lamp examination (Fig. 3) by 16 months of age [65]. These typically cause progressive photophobia beginning in early to mid-childhood. Sun avoidance and the use of dark glasses are common defenses. The corneal crystals themselves do not impair vision, nor does the peripheral retinopathy that occurs early in life [66].

Late ocular complications, however, have serious ramifications. In 8 transplanted patients aged 12–24 years, posterior synechiae, anterior lens crystals, and decreased visual acuity were observed [30]. Dureau et al. evaluated 29 patients and reported photophobia plus retinopathy in 52%, maculopathy in 3 patients, and a flat electroretinogram (ERG) in 3 patients [67]. An evaluation of 172 patients showed anterior chamber involvement that included superficial punctate keratopathy, filamentary keratopathy, band keratopathy, corneal neovascularization, and posterior synechiae with iris thickening [68]. Posterior chamber involvement, assessed in 208 patients, included retinal pigment epithelium mottling, visual field constriction, and impaired rod and cone ERG responses; the frequency of retinopathy was inversely related to the duration of oral cysteamine treatment [69]. In vivo confocal microscopy and anterior segment optical coherence tomography are considered optimal methods for evaluating ocular involvement in cystinosis [70].

### Gastrointestinal manifestations

**Fig. 4:**
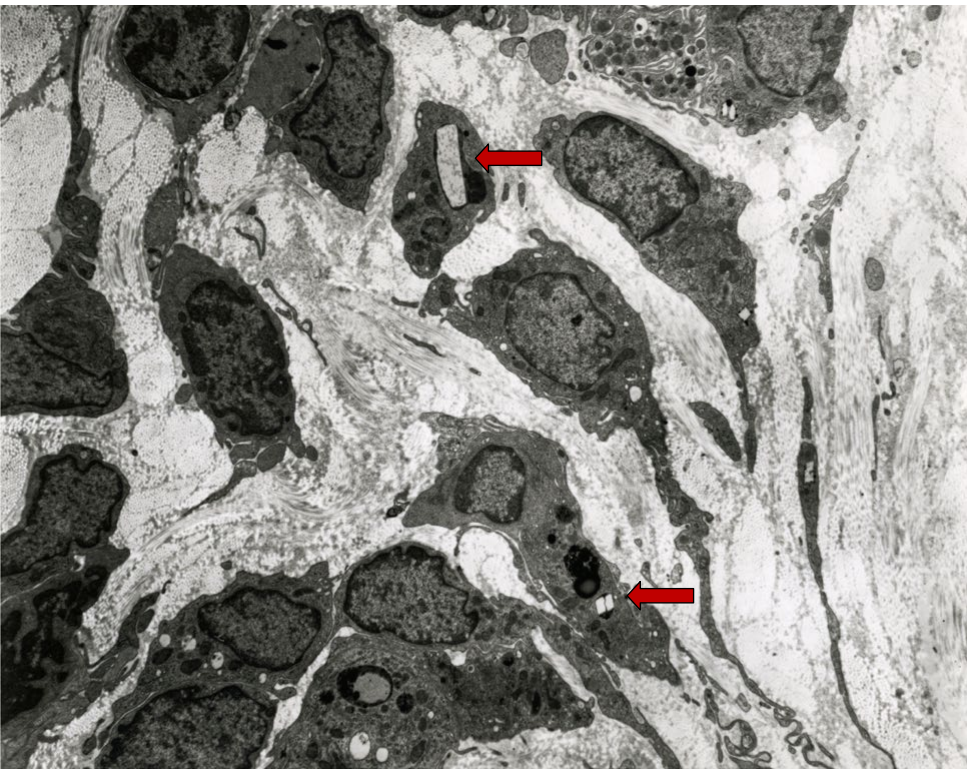
Electron micrograph of an intestinal biopsy showing rectangular cystine crystals (arrows) within the lysosomes of mucosal cells

Gastrointestinal (GI) manifestations of nephropathic cystinosis are extensive and heterogeneous, present early in life, and can persist into adulthood. The etiology can be ascribed to: (1) Fanconi syndrome and its treatments; (2) cystine crystal accumulation in GI tissues (Fig. 4); and (3) cysteamine therapy. The GI symptoms impact growth, nutrition, adherence to treatment, and quality of life, necessitating early recognition and comprehensive management.

#### Effects of kidney disease

In cystinosis, the polyuria of Fanconi syndrome leads to dehydration, extreme thirst, and polydipsia. This causes abdominal distension, premature satiety, nausea, vomiting, and anorexia. At the same time, replacement therapy with bicarbonate, sodium, potassium, citrate, carnitine, phosphate, and vitamin D also enhances nausea, abdominal discomfort, and diarrhea [71]. Elenberg et al. reported that 65 of 70 patients (93%) had prevalent GI issues such as nausea, vomiting, anorexia, abdominal pain, diarrhea/constipation, and failure to thrive at initial presentation. Gastric or jejunal tube feedings occurred in 30% of patients; 7% required continuous or intermittent total parenteral nutrition [72].

Eventually, patients with kidney failure require dialysis and transplantation, which themselves cause GI complications. Peritoneal dialysis is associated with reflux esophagitis, hernias, bowel erosions from catheter pressure, and sclerosing peritonitis while hemodialysis is complicated by nausea, vomiting, intestinal ischemia due to hypotension, and digestive system bleeding due to heparin usage.

#### Direct effects on the GI tract

GI functional abnormalities are well-characterized in the literature. Elenberg et al. reported that, of 35 patients, 15 had gastroesophageal reflux disease, 9 had swallowing dysfunction, 7 had dysmotility, 4 had pseudo-obstruction, 4 had esophagitis, and 2 had gastric or duodenal ulcers [72]. These findings were prevalent early in life. Lower GI symptoms, including diarrhea, constipation, and abdominal pain, are also prevalent. Chronic diarrhea is believed to result from cystine crystal accumulation in the intestinal lining and lamina propria, causing inflammation, malabsorption, and impaired motility. On the other hand, constipation may result from smooth muscle myopathy that decreases peristalsis [73]. Severe GI complications, although rare, have been reported in the literature and include bowel perforation and peritonitis [54]. In a study of 100 adult patients at the NIH Clinical Center, 33 patients had died, including 9 of sepsis, 3 of bowel perforations, and 3 with peritonitis [54].

#### Effects of cysteamine and other medications

Cysteamine is a known ulcerogenic agent that increases gastric acid secretion and gastrin levels. Its side effects, including nausea, vomiting, abdominal pain, and gastric acid hypersecretion, threaten patient compliance with therapy [74]. Proton-pump inhibitors (PPIs), such as omeprazole and esomeprazole, are effective in managing GI discomfort resulting from gastric acid hypersecretion [74]. Despite its significant GI side effects, cysteamine irrefutably mitigates the severity of some GI complications by slowing disease progression. Dohil et al. reported long-term cysteamine treatment was associated with lower crystal concentrations in the GI mucosa; leucocyte cystine levels significantly correlated with the number of crystals in the stomach and duodenum [75].

#### Management

Nutritional and medication-based initiatives can help alleviate symptoms. Supportive therapies, including PPIs, antiemetics, and motility agents, can improve cysteamine treatment adherence. Gastrostomy buttons are particularly beneficial in children with significant feeding difficulties [76].

### Liver and spleen

Hepatic complications range from asymptomatic hepatomegaly to rare, severe conditions such as cholestatic liver disease and non-cirrhotic portal hypertension (NCPH). Over one-third of patients develop hepatomegaly by the age of 5, regardless of the duration of cysteamine use [77].

Histopathology reveals cystine-laden Kupffer cells without classic signs of cirrhosis [78, 79] but sinusoidal congestion, fibrosis, and increased intralobular stellate cells have also been reported [79]. Despite these structural abnormalities, the liver synthetic function is generally preserved unless late-stage disease occurs. Cholestatic liver disease with sclerosing cholangitis is rare and has been reported in 2 post-transplant cystinosis patients. Treatment with ursodeoxycholic acid led to biochemical improvement in one and stabilization in the other [78].

NCPH is a more prevalent complication, driven by nodular regenerative hyperplasia. These patients have too little fibrosis to account for the severity of their portal hypertension [79]. In a 2007 study, 3 of 33 deceased individuals died of portal hypertension; nodular regenerative hyperplasia was documented in 2 [54].

Splenomegaly, often secondary to portal hypertension, occurs relatively frequently; Topaloglu et al. noted that 10 of 21 patients had splenomegaly [73]. While typically asymptomatic, the splenomegaly can progress to hypersplenism, causing thrombocytopenia, leukopenia, anemia [80], and bleeding that can be exacerbated by intrinsic bone marrow defects. Histological findings include foam cells in the red pulp and cystine accumulation in splenic vacuoles. Although splenectomy is rarely indicated, it may be considered in severe cases with symptomatic hypersplenism or recurrent complications [81].

In addition to cysteamine, therapy can include ursodeoxycholic acid for cholestasis [78], porto-systemic shunting for portal hypertension [79], endoscopic banding for esophageal varices [81], and supportive treatments for hypersplenism. Annual liver function tests, ALT, AST, GGT, and ALP lipase studies are recommended, along with regular abdominal imaging if hepatomegaly or splenomegaly are observed. Patients who have both splenomegaly and increased portal vein diameter require close follow-up to prevent progression to fatal complications like portal hypertension with reverse flow and collaterals.

### Bone marrow and hematological defects

The most prevalent hematological complication in cystinosis is pancytopenia. Although generally asymptomatic, some cases are severe, with normochromic, normocytic anemia, thrombocytopenia, and leukopenia [80]. Thrombocytopenia in cystinosis may also result from intraplatelet cystine deposition, which impairs platelet aggregation and increases bleeding risk [80]. Mild leukopenia may enhance vulnerability to infections in patients receiving immunosuppressive therapy following kidney transplantation.

Bone marrow abnormalities involve the accumulation of cystine crystals within macrophages and the extracellular space. This stimulates the reticuloendothelial system, disrupts normal hematopoiesis, and leads to central cytopenias. Most cases present with hypocellular marrow [80].

Bone marrow defects often present later in life, but early-onset cases have been reported in the literature. Bone marrow fibrosis due to kidneyosteodystrophy and elevated parathyroid hormone levels in patients can further impair hematopoiesis [82]. Supportive care includes transfusion and erythropoiesis-stimulating agents. Addressing secondary contributors such as hyperparathyroidism and ensuring adequate nutritional status can further optimize outcomes.

### Cystinosis myopathy and its concomitants

**Fig. 5:**
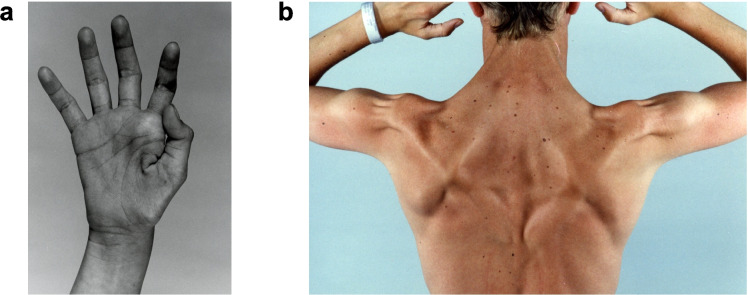
The myopathy of cystinosis. **a** Hand of a post-transplant patient showing wasting of the muscles of the thenar and hypothenar eminences. **b** Severe upper body muscle wasting

Since the first description of muscle disease in nephropathic cystinosis in 1988 [23], there have been several reports of this vacuolar myopathy. Sadjadi et al. described progression from atrophy of the thenar and hypothenar eminences of the hand (Fig. 5a) to the inclusion of distal muscles of both the upper and lower extremities, proximal muscles of the hip, neck, shoulder (Fig. 5b), and/or muscles involved in speech, mastication, swallowing, and ventilation [83]. There exists, however, considerable heterogeneity in the muscle involvement of cystinosis, even amongst siblings. This highlights the potential influence of genetic and/or environmental factors in addition to primary *CTNS* mutations on the muscle disease of cystinosis.

#### Skeletal muscle involvement

Skeletal muscle biopsies are characterized by autophagic vacuoles and acid phosphatase-positive areas, indicating lysosomal involvement, as well as cystine crystal deposition within the perimysium [24, 84]. Since autophagic vacuoles do not appear in every biopsy, their absence should not rule out the diagnosis of cystinosis myopathy. Nevertheless, the amount of cystine crystal accumulation in muscle correlates with the severity of muscle involvement [24]. Although steroid treatment used for immunosuppression for kidney transplantation and chronic kidney disease itself can contribute to loss of muscle mass and strength, the impact of cystine accumulation on muscle pathology is widely recognized. In one study of 76 adult and pediatric patients, those with nephropathic cystinosis had a mean grip strength z-score of − 2.1 (SD, 1.1); this was lower than for patients with chronic kidney disease due to other causes [85].

Some pediatric cases of distal myopathy have been noted [86]. In these children, skeletal muscle involvement was subtle and generally included difficulties with fine motor skills, decreased physical endurance, and mild axial weakness of neck flexors and/or abdominal rectus muscles [86]. By adolescence or adulthood, muscle wasting becomes more apparent, especially in the distal limbs [85, 86]. Strikingly, myopathic changes are detected even when overt weakness is not present or noticed by the patient. In a study of 7 patients with cystinosis, all showed signs of myopathy on electromyography (EMG) even though only two complained of distal muscle weakness [84]. In another study, all 55 patients (ages 2.8–41.3 years) underperformed on a mechanography platform [86].

#### Swallowing dysfunction and dysphagia

Dysphagia and oral motor dysfunction leading to difficulties with feeding and swallowing can present in early childhood. In a study of 22 children and adolescents, parents of 20 patients noted early feeding and oral motor problems [87]. Ten of the 20 had difficulty transitioning to baby food and table food; 5 required occupational therapy for feeding or oral motor problems, 7 required a G-tube for supplemental nutrition, and 6 chewed foods for unusually long periods of time before swallowing. Gagging, choking, vomiting, slow eating, swallowing more than once for a single bite of food and difficulty chewing and articulating speech were notable in these young patients. Dysphagia and swallowing difficulties are common and increase the likelihood and severity of aspiration pneumonia, other pulmonary infections, and choking. Indeed, Sonies et al. reported episodes of aspiration prior to death in 9 of 20 deceased cystinosis patients [26]. Oropharyngeal dysfunction is found broadly across cohorts [25]. Even without clear complaints of dysphagia, cystinosis patients require more bites, mastication cycles, number of swallows, and time to finish eating [88]. Thickening foods to increase sensation, pureeing to ease swallowing, and placing food closer to the back of the tongue may help to overcome defects in the oral and pharyngeal phases of swallowing. Muscle training may also mitigate effects; Sadjadi et al. found that 5 weeks of using a handheld device to improve the activation of muscles involved in swallowing and clearing the trachea improved swallowing in patients with rather severe dysphagia [83].

#### Pulmonary dysfunction

Weakness of respiratory muscles, such as the diaphragm and intercostal muscles, can impair lung mechanics, reduce the ability to cough, and increase the risk of sleep apnea and respiratory failure. Across all ages of individuals with cystinosis, the primary cause of pulmonary dysfunction is muscle weakness. This myopathy affects both inspiratory and expiratory muscles, limiting lung volumes and coughing efficiency [89]. It often begins subtly in childhood and becomes clinically significant in adulthood, increasing in severity with age.

One study found small airway disease in 12 of 15 children aged 3–15 years using impulse oscillometry (IOS) testing, even in the absence of clinical respiratory symptoms, significant chest complaints, or chest X-ray abnormalities [90]. A cohort of 12 adults lacking compliant cystine-depleting therapy for at least 17 years found that key pulmonary function metrics, such as the mean FVC, FEV1, and TLC, were significantly reduced at 58%, 57%, and 66% of predicted, respectively. These measurements corresponded with significant reductions in mean inspiratory and expiratory pressures [89]. Such findings support the role of weakened respiratory muscle in the pathophysiology of pulmonary dysfunction in nephropathic cystinosis.

#### Monitoring of cystinosis myopathy

Because of the life-long progression of muscle weakness in nephropathic cystinosis, regular evaluation and tracking of chronic symptoms, including those related to dysphagia and respiration, are essential. Electroneuromyography (ENMG) is recommended at baseline when signs of muscle weakness and wasting first appear but is not advised for routine follow-up unless new symptoms emerge or muscle versus peripheral nerve involvement needs to be differentiated [91]. Instead, clinical history-taking should focus on patient-reported difficulties such as chewing, aspiration, dysphagia, excess saliva, weight loss, prolonged mealtimes, and respiratory symptoms, which can guide further diagnostic evaluations. Diagnostic evaluations such as modified barium swallow tests, oral sensorimotor exams, and video fluoroscopy are necessary for identifying dysphagia and other swallowing dysfunctions. Spirometry should also be performed to detect pulmonary restriction as soon as weakness in skeletal muscle groups is evident. Importantly, measures of arterial oxygen tension and hemoglobin oxygen saturation typically remain normal until respiratory weakness and hypoventilation become severe [90]. Cysteamine may be able to delay muscle involvement in cystinosis, but not fully prevent it [85].

### Cardiovascular complications

Due to kidney failure, individuals with cystinosis often experience renin-induced hypertension if they have not had their native kidneys removed. In addition, patients who have undergone transplant can still develop calcifications of blood vessels, most often in the coronary arteries [32]. Risks for atherosclerosis and cardiovascular disease are comparable to those for others with KF [92]. Hypercholesterolemia often begins in early childhood; these levels improve with transplantation but remain elevated above those of patients with other causes of glomerular insufficiency alone [93]. Patients should be monitored in adulthood for signs of myocardial ischemia and infarction.

### Neurologic complications

Individuals with cystinosis generally do not have severe intellectual deficits or neurologic findings, but some distinct patterns have emerged. Full-scale IQ levels fall within the low-normal to normal range, although these scores may be lower than would be expected relative to the IQ levels of siblings or parents [94]. Deficits in visual-spatial processing can affect visual-motor integration, mental rotation, and processing of complex visual stimuli [95, 96]. Besouw et al. found that verbal IQ, i.e., related to language and comprehension, tended to be higher than performance IQ, i.e., related to non-verbal spatial processing and integration [97]. Tests specific for visual-motor integration showed significant impairment in individuals with cystinosis. For example, a cohort of 23 patients with cystinosis struggled more than control participants when asked to identify an object without any visual input; this effect was more pronounced with age [95, 96]. Electrophysiologic studies have found increased amplitude responses to visual stimuli [98], posing problems for students in math and spelling, which rely heavily on spatial reasoning and manipulation [99]. Deficits in executive functioning and attention can also impact organizational abilities [97, 100]. Individuals with cystinosis struggle with attention and behavior issues [101]. Impaired auditory sensory processing and memory, apparent in electrophysiologic studies, could contribute to attentional difficulties [99, 102, 103]. Heterozygotes for cystinosis have evidence of similar cognitive deficits [99]. Early and consistent treatment with cysteamine correlates with higher IQ scores [104, 105].

Cortical and sub-cortical cerebral atrophy have been reported [28, 104, 106–108]; Nichols et al. found that 10 of 11 children with cystinosis had cortical atrophy on MRI, which correlated with impairment in cognitive tasks involving visual memory [106]. A study of 17 adult patients found that 72% exhibited cortical atrophy and 67% showed ventriculomegaly [104]. Ventricular dilation and calcification have also been reported [109]*.*

Several cases of idiopathic intracranial hypertension (IIH) have been reported [31, 110, 111]. Dogulu et al. found eight patients with evidence of papilledema but without concurrent neuroimaging or CSF abnormalities [31]. It was speculated that IIH in cystinosis could be secondary to thrombotic risk from kidney disease or impaired CSF reabsorption in arachnoid villi due to cystine crystal deposition [31, 110]. In another study of 8 children with cystinosis, 4 had intracranial hypertension with papilledema and increased CSF pressure, and two required ventriculoperitoneal shunting [111]. Monitoring for papilledema or other evidence of changes to the optic nerve or disc should be conducted through yearly, standard fundoscopic examinations [31, 110, 111].

Cystine crystal deposition has been noted throughout the brain; Ross et al. documented nonabsorptive hydrocephalus associated with cystine deposits in the choroid plexus, leading to disrupted cerebrospinal fluid dynamics [107, 112]. A progressive cystinosis encephalopathy may present with cognitive decline or stroke-like symptoms, recurrent seizures, as well as impaired cognition and motor deficits [104, 109, 113]. Calcifications and cystic necrosis occur in some symptomatic patients, usually in the basal ganglia, periventricular regions, and internal capsule [28, 101, 107, 112, 113]. White matter hyperintensities and demyelination have been observed in the corpus callosum, periventricular regions, and parietal lobe; this could alter pathways involved in spatial reasoning [28, 105, 114, 115]. Servais et al. have provided an in-depth review of imaging in cystinosis [116]. Early intervention with cysteamine has been repeatedly noted to mitigate any neurodegenerative effects, particularly if patients have begun cysteamine before the age of 2 [99, 108, 109].

### Psychosocial well-being

Limited research has been conducted on the mental health and psychosocial well-being of patients with cystinosis. A hospital anxiety and depression scale questionnaire of 23 adults with cystinosis found that 18 met the criteria for high levels of anxiety and/or depression [117]. Patients with cystinosis have consistently lower health-related quality of life than other subjects [118], related to struggles with chronic fatigue and short stature that prompted bullying [117, 118]. Side effects from cysteamine treatment, such as body odor, halitosis, and nausea, further affected the social lives of those with cystinosis [117–119], prompting adolescents to hide their disease or avoid taking cysteamine [117]. Caregivers can also experience decreased quality of life, anxiety, and depression [120]. Individuals with cystinosis, however, are extremely resilient and exhibit coping strategies involving community support [117]. A steering committee of experts familiar with cystinosis outlined areas of concern when transitioning to adult care; behavioral issues and attentional deficits and fears about disease progression contribute to stress and anxiety [119]. Early, sustained psychosocial support is important for all cystinosis patients.

### Life expectancy

**Fig. 6:**
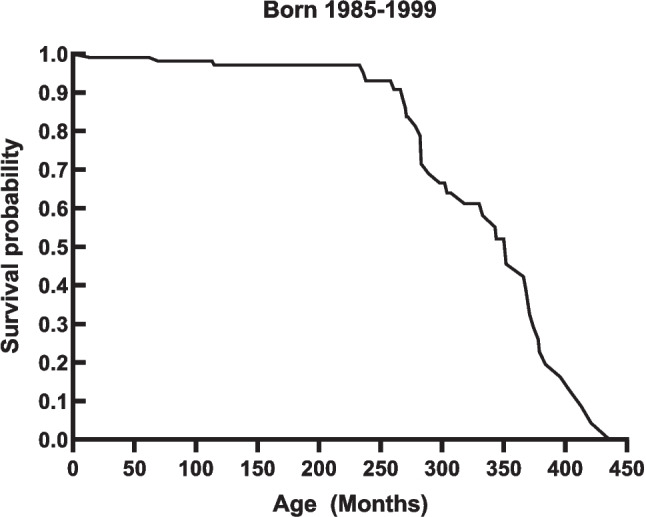
Kaplan-Meier plots for survival of NIH cystinosis patients born between 1985 and 1999

A 2007 article found that 33 adults with cystinosis had died at a mean age of 28.5 years [54]. Our current Kaplan-Meier analysis of 110 patients seen at the NIH Clinical Center and born between 1985 and 1999 indicates that 50% have succumbed at 28.8 years of age (Fig. 6). Since this plot does not account for individuals born before 1985, no survivors past age 39 are included. However, we and others are aware of individuals with cystinosis who are alive in their forties or even fifties [40, 121]; this requires early, diligent, and consistent initiation of cysteamine therapy, and regular monitoring of WBC cystine levels, which is not available to all individuals worldwide [121, 122]. With improvements in newborn screening, it is our hope that those with cystinosis can be identified and treated early to extend life expectancy and time to the first transplant.

## Recent advances

### Oral cysteamine therapy

Since the FDA approved oral cysteamine, additional publications have documented its benefits [26, 53, 54, 123, 124]. In fact, many individuals with cystinosis reach adulthood free of significant nonrenal complications of the disease. However, as a free thiol, cysteamine smells like rotten eggs and creates halitosis [125, 126] and a foul odor on the skin and in sweat. For those on higher doses of cysteamine, GI upset and vascular lesions have occurred [127]. As previously discussed, the unpleasant side effects of oral cysteamine cause reduced compliance, especially in adolescents [128]. Because non-kidney complications of cystinosis progress despite kidney replacement [129], oral cysteamine treatment should continue after transplantation.

More tolerable cysteamine preparations have been pursued, including altered forms of drug delivery using gastrointestinal infusion and delayed enteric-release preparations of the drug [130]. A comparison of the delayed release preparation with Cystagon^®^ showed non-inferiority with respect to leucocyte cystine depletion [131]. The delayed-release preparation was approved by the FDA in 2013 as Procysbi^®^, with the suggestion to take it with meals. Benefits of this formulation include increased quality of life[132], although Procysbi^®^ costs nearly 100 times as much as Cystagon^®^ [133]. In our experience, approximately half of patients use Cystagon^®^ and half use Procysbi^®^.

### Kidney function and compliance with oral cysteamine therapy

In 2022, Niessl et al. evaluated data from 52 cystinosis patients followed in Germany between 1997 and 2020; the estimated glomerular filtration rate (eGFR) was greater in early-treated patients than in later-treated patients [134]. In 2015, Nesterova et al. created a cysteamine compliance score based upon the duration of treatment and the extent of leucocyte cystine depletion; the greater the compliance score, the longer glomerular function was preserved [135]. In contrast, there was no relationship between the compliance score and tubular function. It has long been accepted that the Fanconi syndrome of cystinosis cannot be reversed, but in 2022 Hohenfellner et al. showed that it could be prevented. Four infants with good compliance with oral cysteamine starting before 2 months of age not only maintained normal eGFR for years, but also maintained normal serum potassium, bicarbonate, phosphate, and calcium levels without supplementation [136].

**Fig. 7:**
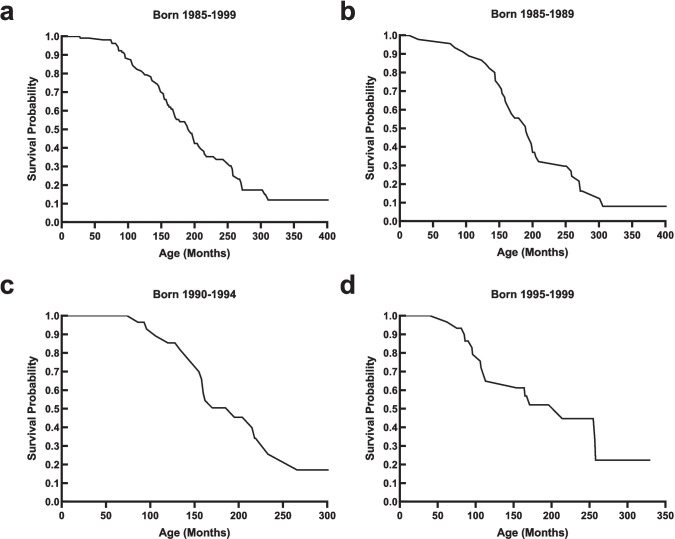
Kaplan-Meier plots for kidney survival of NIH cystinosis patients. **a** Patients born 1985–1999. **b** Patients born 1985–1989. **c** Patients born 1990–1994. **d** Patients born 1995–1999

These findings give hope that initiating cysteamine treatment in the neonatal period could prevent both the glomerular and tubular damage of cystinosis. Currently, however, irreversible damage has occurred by the time of diagnosis, and kidney failure eventually occurs. The age of kidney failure for cystinosis patients born between 1985 and 1999 and followed at the NIH is shown in a Kaplan-Meier plot in Fig. 3. Fifty percent of individuals required dialysis or transplantation by 15.83 years of age (190 months) (Fig. 7a); this was 80 months later than reported for the natural history of patients not receiving cysteamine, i.e., 9.2 years or 110 months [14]. When divided into 5-year periods of birth date, there was no discernible trend, with a mean age at kidney failure of 189 months for patients born between 1985 and 1989 (Fig. 7b), 170 months for those born 1990–1994 (Fig. 7c), and 196 months for those born 1995–1999 (Fig. 7d). These data suggest that the glomerulus-sparing efficacy of cysteamine has not increased over the past decades, probably because the rate-limiting issue is a delay in diagnoses and cysteamine treatment.

### Advances in topical cysteamine therapy (eyedrops)

The safety and efficacy of cysteamine eyedrops in dissolving corneal cystine crystals [21, 22] were confirmed in several single case reports and in one published series [137]. However, some studies found no improvement in photophobia or corneal crystal density [138, 139], suggesting that compliance plays a major role in determining outcome. In 2012, cysteamine hydrochloride (0.44%) eyedrops were approved by the FDA as Cystaran^®^, with instructions to be administered every hour while awake, but that frequency was dictated only by the fact that the pivotal clinical trial employed such a regimen. In our experience, administration of Cystaran^®^ eyedrops several times a day (e.g., 4–6 times) dissolves corneal crystals.

Nevertheless, a gel formulation of cysteamine 0.55% was developed to be delivered 4 times per day and showed long-term safety and superiority when compared with a lower concentration (0.1%) of cysteamine hydrochloride [140]. In 2020, this formulation was approved by the FDA as Cystadrops^®^. Other preparations under evaluation have included a nanowafer impregnated with cysteamine [141] and a sustained-release gel eyedrop [142].

### Newborn screening

Early initiation of cysteamine significantly delays kidney failure, reduces the risk and severity of systemic complications, and improves growth and quality of life in patients with nephropathic cystinosis. Studies demonstrate that starting treatment before 2 years of age preserves kidney function more effectively than later initiation. Because cystinosis today is often not diagnosed until patients are 1–1.5 years of age, significant kidney tubular and glomerular damage has already occurred at this point. Hence, early diagnosis of cystinosis through newborn screening (NBS) is critical to prevent irreversible damage and enable timely cysteamine treatment [143].

Unfortunately, no biochemical markers of cystinosis are detectable in whole blood in the neonatal period, so the current NBS method of tandem mass spectrometry is unsuitable. Consequently, Hohenfellner et al. conducted an NBS pilot study in Germany (2018–2019) integrating the already existing newborn screening framework with first-tier, high-throughput molecular genetic screening targeting the most common disease-causing variants in the screened population. Second-tier screening involved sequencing the entire gene for 101 pathogenic variants. Of 250,000 screened newborns, two affected individuals were detected. Cysteamine therapy was initiated in a 3-week-old newborn homozygous for the 57-kb deletion; this prevented all kidney complications at 16 months of age [143]. Although based on a single case, this outcome underscores the transformative potential of early detection in improving patient outcomes.

Efforts to harmonize NBS programs are underway in Europe and the USA and aim to standardize practices while expanding access. However, the prevalence and types of *CTNS* mutations vary significantly by region. For example, the 57 kb deletion is common in Europe and North America but absent in Turkey and Egypt, where the c.681G>A (p.E227E) and c.829 dup (p.T277 NfsX19) variants, respectively, predominate. This emphasizes the need for region-specific panels for effective screening [143].

### Pathogenesis and developing therapies

Historically, cystinosis has been understood as a lysosomal storage disorder caused by cystine accumulation and subsequent lysosomal dysfunction. However, recent research has uncovered a broader range of pathophysiologic mechanisms contributing to disease progression [144–147].

Cystinosin deficiency leads to impaired lysosomal clearance and disrupted endolysosomal trafficking, which in turn contributes to defective autophagy and altered proteostasis [144, 146, 147]. This impairment results in increased apoptosis, particularly in renal proximal tubular cells, exacerbating kidney dysfunction [145]. Additionally, altered calcium signaling may play a role in defective endolysosomal trafficking, further affecting cellular homeostasis [146]. Dysregulated mTORC1 and TFEB signaling exacerbate these autophagic defects, prompting research into the use of autophagy modulators as potential therapeutic strategies [144, 146].

Mitochondrial abnormalities are another hallmark of cystinosis, with studies demonstrating reduced ATP production and evidence of metabolic imbalances [144, 146, 148]. The sequestration of cystine within lysosomes may deplete cytosolic cysteine, which is essential for glutathione synthesis, leading to impaired redox homeostasis [144, 146]. Increased oxidative stress and energy depletion contribute to cellular dysfunction and apoptosis, highlighting the need for antioxidant therapies. Indeed, cysteamine usage coupled with repurposed medications has been examined in cystinosis. Specifically, drugs that reduce oxidative stress and improve lysosomal function are of interest; however, these combinations have yet to be standardized [149, 150].

Inflammasome activation, macrophage infiltration, and cytokine production have been documented in cystinosis, indicating that chronic inflammation plays a key role in disease pathogenesis [147]. Persistent inflammatory responses contribute to interstitial fibrosis, further accelerating kidney damage and systemic complications [145, 147]. A deeper understanding of these interconnected pathophysiologic mechanisms will facilitate the development of targeted therapies beyond cysteamine, optimizing treatment approaches to mitigate disease progression [146].

Alternative approaches to oral cysteamine therapy have been proposed for cystinosis [82, 146, 151, 152]. Repurposing of already approved drugs has been explored, for example, disulfiram, a drug traditionally used to treat alcohol dependence, can lower cystine levels in cystinotic cells, but has side effects that limit its use in cystinosis [151, 153]. Translational readthrough agents, such as Geniticin and ELX- 02, which allow translational readthrough of premature stop codons in mRNA to produce the full-length protein, have been explored for *CTNS* missense mutations. Unfortunately, Geniticin, an aminoglycoside antibiotic, leads to ototoxicity and nephrotoxicity through interaction with mitochondrial ribosomes [154]. ELX- 02, a novel aminoglycoside analog, avoided serious adverse events in preclinical models [155] and Phase 1 clinical trials [156]. Phase 2 studies showed therapeutic levels could be achieved in patients. However, its efficacy is limited to patients with at least one nonsense mutation who represent only 15% of cystinosis cases [7] and it requires repeated dosing to maintain therapeutic effects.

Synthetic mRNAs, which transiently express functional cystinosin, have demonstrated beneficial and sustained impacts on proximal tubular resorptive function, proteinuria, and kidney morphology in zebrafish after treatment [157]. Although benefits of mRNA *CTNS* delivery are mutation-independent, efficient delivery to kidney cells and expression of the mRNA remain difficult [155, 157]. Cystinosin-LKG, the alternatively spliced isoform of cystinosin, has a broader localization that could be leveraged to reduce cystine accumulation, restore lysosomal function, and lower apoptosis rates in cystinotic proximal tubule cells [155]. Autologous hematopoietic stem cell transplants have been performed in a few cystinosis patients based upon the premise that tunneling nanotubes transport lysosomes bidirectionally between the stem cells and the patient’s parenchymal cells [158].

#### Disparities in care

In developing economies, median native kidney survival is 6.4 years shorter than in developed economies [159]. This gap is exacerbated by insufficient access to supportive therapies like dialysis and transplantation; only 59% of centers in developing economies provide access to kidney transplantation. Moreover, only 54% of patients in those economics have access to oral cysteamine therapy, and there is limited access to cysteamine eye drops, supportive therapies, and standard diagnostic and monitoring tools, all of which are essential for the prevention of disease progression and extended lifespan [159]. One initiative to address this healthcare disparity has been instituted in South Africa, which provides cysteamine powder as an affordable alternative [160]. Advocating for cysteamine’s inclusion in essential medicines lists is critical [161].

## Conclusions

The natural history of kidney disease in nephropathic cystinosis, involving Fanconi syndrome in infancy and glomerular failure at approximately 10 years of age, has been well-documented. Kidney transplantation and cystine-depleting therapy with oral and topical cysteamine have transformed cystinosis from a universally fatal childhood disorder into a treatable chronic disease, with many adults achieving normal lives. This still requires a team of professionals to manage the transition of care from the pediatric to the adult setting. Despite medical interventions, the age at kidney failure has remained ~ 16 years for patients born between 1985 and 2000, and the age at death for those individuals still approximates 29 years. These sobering facts require the pursuit of: (1) Newborn screening to diagnose and treat infants in the first week or two of life; (2) More tolerable preparations of cysteamine; and (3) Gene therapy approaches that correct the defective lysosomal cystine transport in parenchymal cells.

## Supplementary Information

Below is the link to the electronic supplementary material.

Graphical abstract (PPTX 366 KB)

## Data Availability

The datasets generated and/or analyzed during the current study are not publicly available because they contain PII (birthdates) but are available in a de-identified fashion from the corresponding author on reasonable request.
