# Supplementary material for: Long-term outcomes in nephropathic cystinosis: a review
Source: Pediatr Nephrol. 2025 May 14;41(2):277–96. doi: 10.1007/s00467-025-06790-6 (PMC12727779; doi:10.1007/s00467-025-06790-6)
Supplement: Supplementary file 1 — Graphical abstract (PPTX 366 KB) [file 467_2025_6790_MOESM1_ESM.pptx]

## Slide 1
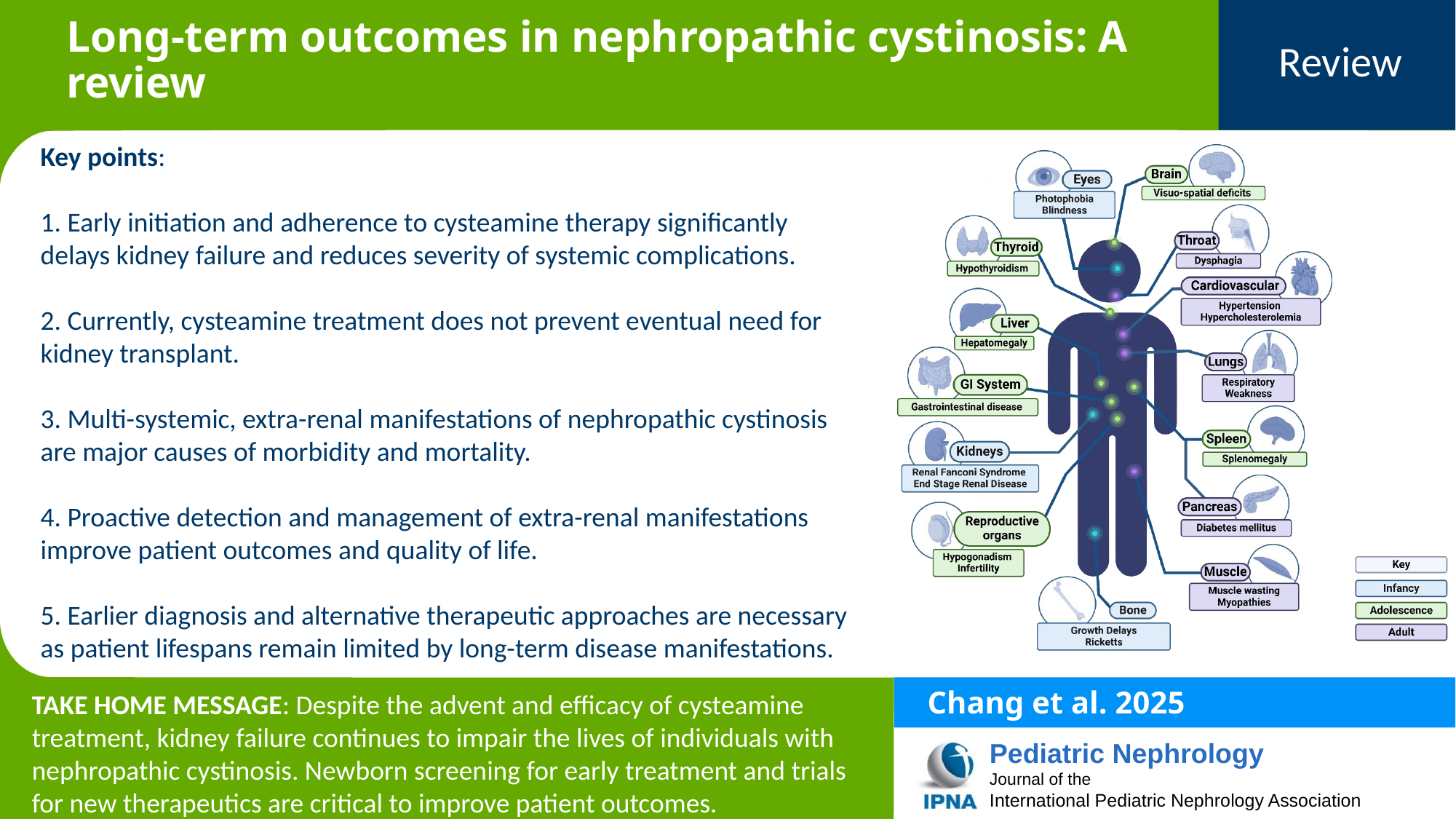

Long-term outcomes in nephropathic cystinosis: A review
Key points:
1. Early initiation and adherence to cysteamine therapy significantly delays kidney failure and reduces severity of systemic complications.
2. Currently, cysteamine treatment does not prevent eventual need for kidney transplant.
3. Multi-systemic, extra-renal manifestations of nephropathic cystinosis are major causes of morbidity and mortality.
4. Proactive detection and management of extra-renal manifestations improve patient outcomes and quality of life.
5. Earlier diagnosis and alternative therapeutic approaches are necessary as patient lifespans remain limited by long-term disease manifestations.
Chang et al. 2025
TAKE HOME MESSAGE: Despite the advent and efficacy of cysteamine treatment, kidney failure continues to impair the lives of individuals with nephropathic cystinosis. Newborn screening for early treatment and trials for new therapeutics are critical to improve patient outcomes.
